# Differential expression of 2IgB7-H3 and 4IgB7-H3 in cancer cell lines and glioma tissues

**DOI:** 10.3892/ol.2015.3611

**Published:** 2015-08-14

**Authors:** ZHENXIN WANG, JIANFENG YANG, YANBO ZHU, YUN ZHU, BIN ZHANG, YINGHUI ZHOU

**Affiliations:** 1 Department of Oncology, The First Affiliated Hospital of Soochow University, Suzhou, Jiangsu 215004, P.R. China; 2 Cyrus Tang Hematology Center, Soochow University, Suzhou, Jiangsu 215123, P.R. China; 3 Department of Cerebral Surgery, The First Affiliated Hospital of Soochow University, Suzhou, Jiangsu 215004, P.R. China; 4 Department of Biochemistry and Molecular Biochemistry, School of Medicine, Soochow University, Suzhou, Jiangsu 215123, P.R. China

**Keywords:** B7-H3, monoclonal antibody, expression, glioma

## Abstract

B7-H3 protein is an important tumor antigen, but the expression of its isoforms, 4IgB7-H3 and 2IgB7-H3, in tumor tissues remains unknown due to the lack of specific monoclonal antibodies (mAbs). In the present study, a mAb (9C3) specifically recognizing 2IgB7-H3, but not 4IgB7-H3, was prepared. Using 9C3 and a previously prepared mAb (4H7) that recognizes 4IgB7-H3 and 2IgB7-H3, the differential expression of 2IgB7-H3 and 4IgB7-H3 was analyzed in a variety of tumor cell lines by flow cytometry. It was found that 4IgB7-H3 had a more broad spectrum of expression among the cell lines compared with 2IgB7-H3. The expression of the two isoforms was further examined in glioma tissues using reverse transcription-polymerase chain reaction and immunohistochemistry techniques. The data revealed that 2IgB7-H3, but not 4IgB7-H3, was specifically expressed in glioma. Taken together, these results demonstrated for the first time that 2IgB7-H3 is a valuable biomarker for the diagnosis of glioma.

## Introduction

The human B7-H3 molecule, which consists of two isoforms, 4IgB7-H3 and 2IgB7-H3, belongs to the immunoglobulin family. The structure of the two isoforms is extremely similar with the exception that 4IgB7-H3 has an extra IgC-IgV immunoglobulin domain in the extracellular region (1–3). The B7-H3 molecule is expressed at a low level in normal tissues, but its expression is increased in several types of tumor tissues, including lung, colorectal kidney and breast cancer, neuroblastoma and melanoma (4–10). It has been suggested that B7-H3 is a tumor-associated antigen (11–13). Clinical studies have shown that B7-H3 expression is associated with tumor metastasis and is correlated with a poor prognosis (14,15). Roth *et al* studied 338 prostate cancer samples by immunohistochemistry and found the expression of B7-H3 in all these samples. Notably, it was shown that cancer patients with higher B7-H3 expression in tumor tissues had a significantly higher recurrence rate following curative surgery than those with lower B7-H3 expression levels (14). However, Wu *et al* found that a high expression level of B7-H3 in stomach cancer was correlated with a better prognosis (15). The discrepancy between these studies may result from the examination of total B7-H3 rather than the individual isoforms. At present, there is no suitable method to discriminate 2IgB7-H3 from 4IgB7-H3.

In the present study, the expression of the B7-H3 isoforms was examined in different cell lines using the two different monoclonal antibodies generated at the Department of Biochemistry and Molecular Biochemistry, School of Medicine, Soochow University (Suzhou, China). One antibody (9C3) specifically binds 2IgB7-H3, the other (4H7) recognizes the two isoforms of B7-H3. The expression of B7-H3 isoforms in human gliomas was also examined.

## Materials and methods

### 

#### Cell lines

The mouse myeloma SP2/0 cell line, the human kidney endothelial 293T, HUVEC, HK-2, PODO, MC and EAhy926 cell lines, and the human tumor A549, H446, H460, H1299, SPCA-1, Raji, Daudi, K562, Jurkat, 8266, U266, THP-1, SHI-1, U937, HL60, Caco-2, Colo320, CW-2, SW480, LS174T, U251, SHG-44, 767, HEP-2, HepG2, AGS, SW1990, Y79, HeLa, SIHA, M435, M231, HO-8910, SK-BR-3 and WI-38 cell lines were originally obtained from the American Type Culture Collection (Rockville, MD, USA). The 2IgB7-H3-transfected L929 cell line (L929/2IgB7-H3), the 4IgB7-H3-transfected L929 cell line (L929/4IgB7-H3) and the empty vector-transfected L929 cell line (L929/mock) (16), plus L929/B7-H1, L929/B7-H2, L929/PDL1, L929/PDL2, L929/CD209, L929/OX40, L929/LIGHT and L929/HVEM were constructed in the Department of Biochemistry and Molecular Biochemistry, School of Medicine, Soochow University. Peripheral blood mononuclear cells (Suzhou Central Blood Bank, Suzhou, China) and umbilical cord blood (The First Affiliated Hospital of Soochow University) were isolated by Ficoll-Hypaque (Shanghai Second Chemistry Factory, Shanghai, China) gradient centrifugation. The cell lines were cultured in RPMI-1640 medium (Gibco BRL, Grand Island, NY, USA) or standard Dulbecco's modified Eagle's minimal essential medium (Life Technologies, Grand Island, NY, USA), supplemented with 10% fetal calf serum (Hyclone, Logan, UT, USA), 100 U/ml penicillin, 100 mg/ml streptomycin and 2 mM L-glutamine. The cells were cultured in a 5% CO_2_, 37°C incubator.

#### Mice and reagents

Female BALB/c mice were purchased from the Department of Experimental Animals (Shanghai Institute of Biological Products, Ministry of Health of China, Shanghai, China). HAT and HT media were purchased from Sigma-Aldrich (St. Louis, MO, USA). Recombinant human 4IgB7-H3/Fc chimera (cat. no. 2318-B3/CF; 1:100), recombinant human 2IgB7-H3/Fc chimera (cat. no. 1949-B3; 1:100), rhGM-CSF (cat. no. 215-GM/CF; 1:2,000), rhIL-4 (cat. no. 204-IL/CF; 1:1,000), CD3-FITC/PE (cat. no. FAB100F; 1:50), CD14-FITC/PE (cat. no. FAB3832P; 1:50), CD19-FITC/PE (cat. no. FAB4867P; 1:50) and CD56-FITC/PE (cat. no. FAB2408P; 1:50) were purchased from R&D Systems (Minneapolis, MN, USA). Goat anti-mouse IgG-PE (cat. no. IM0855; 1:200) and IgM-FITC (cat. no. 6602434; 1:200) and secondary antibodies (cat. no. M0855; 1:200) were from obtained Beckman Coulter Inc. (Indianapolis, IN, USA). DAPI fluorescent nuclear stain was purchased from Roche Diagnostics (Mannheim, Germany). Mouse monoclonal antibody (mAb) 4H7 with the ability to recognize human 2IgB7-H3 and 4IgB7-H3 isoforms was previously generated in our laboratory (16).

#### Generation of mouse anti-human 2IgB7-H3 mAbs

Female BALB/c mice (6–8 weeks old) were immunized with human 293T cells as an immunogen by intraperitoneal injection of 1×10^7^ cells per mouse. The spleen B cells of the mice were fused with SP2/0 by conventional methods. Hybridoma cells were screened with L929/4IgB7-H3-, L929/2IgB7-H3- and L929/mock-transfected cells by flow cytometry. Positive clones were selected for using a limiting dilution technique.

#### Specificity of the 2IgB7-H3 mAb 9C3

To determine the specificity of the 2IgB7-H3 mAb 9C3, the L929/B7-H1, L929/B7-H2, L929/PDL1, L929/PDL2, L929/CD209, L929/LIGHT, L929/OX40 and L929/HVEM cells were incubated with mouse anti-human 2IgB7-H3 mAb 9C3 and 4H7 mAb (2 µg/ml) for 30 min at 4°C and then washed with phosphate-buffered saline (PBS). PE-labeled goat anti-mouse IgG (1:500 dilution) as secondary antibody was added for another 30 min at 4°C, followed by further washing with PBS. All the transfected cells were analyzed by flow cytometry.

#### Flow cytometry analysis

The cells (1×10^6^ cells/test) were incubated with 9C3 or 4H7 mAb for 30 min at 4°C and washed. PE-labeled goat anti-mouse IgG was added as secondary antibody and incubated for another 30 min at 4°C. The cells were analyzed by flow cytometry and the Expo32 Multicomp software (Beckman Coulter Inc., Brea, CA, USA).

#### Clinical samples and immunohistochemistry

Tissue samples were obtained from the First Affiliated Hospital of Soochow University. The study was approved by the Ethics Committee of Soochow University and all patients provided written informed consent. All procedures were performed according to standard practice. The fresh human glioma tissues were fixed with 10% neutral formalin, embedded in paraffin and processed as 5-µm sections. Four or five adjacent ribbons were collected for histopathological analysis (hematoxylin and eosin stain) and immunohistochemical staining. The histopathological diagnosis for tumor tissues and non-tumor tissues was formed according to cellular morphological changes and tissue architecture using established criteria (17). The biotin-streptavidin complex method was used for the detection of B7-H3 protein using a commercial immunoperoxidase staining kit (Maixin-Bio Co., Ltd., Fuzhou, China). B7-H3-positive cells were characterized by a clear brown color in the cytoplasm and cell membrane. Specimens with >10% positive cells were graded as positive.

#### Reverse transcription-polymerase chain reaction (RT-PCR) analysis of B7-H3 expression in normal and glioma tissues

Normal and glioma tissues were homogenized and total RNA was extracted using TRIzol reagent (Invitrogen Life Technologies, Carlsbad, CA, USA). cDNA was obtained by RT. The mRNA expression of B7-H3 isoforms was analyzed by semi-quantitative PCR using two pairs of primers as follows: Forward, 5′-CTCACGAAGCAGGTGAAGCTGCC-3′ and reverse, 5′-ACCTACAGCTGCCTGGTGCGCAA-3′ for 4IgB7-H3; and forward, 5′-TGTGATGGTGACAGAGCCGTGC-3′ and reverse, 5′-ATGCTGCGTCGGCGGGGCA-3′ for 2IgB7H3. β-actin was used as a control.

#### Statistical analysis

Statistical analysis was performed using SPSS 13.0 statistical software (SPSS, Inc., Chicago, IL, USA). Values are presented as the mean ± standard deviation, and the differences between groups were assessed by Student's t-test. P<0.05 was considered to indicate a statistically significant difference.

## Results

### 

#### Establishment and characterization of novel mouse anti-human 2IgB7-H3 mAbs

**Figure 1.**
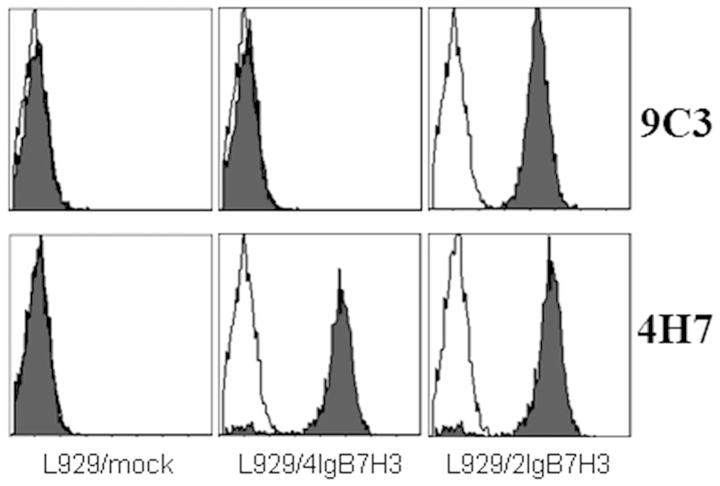
Characterization of the monoclonal antibodies 9C3 and 4H7. 9C3 antibody recognized L929/2IgB7-H3 cells, but not L929/4IgB7-H3 and L929/mock transfectants. 4H7 antibody recognized L929/2IgB7-H3 and L929/4IgB7-H3, but not L929/mock transfectants.

Balb/c mice were immunized with 293T cells and the splenocytes were fused with murine myeloma SP2/0 cells by standard procedures. Subsequent to multiple subcloning and repeated screening, one hybridoma (termed 9C3), which specifically binds to human 2IgB7-H3-transfected cells, was collected and further characterized. The isotope of 9C3 was mouse IgG with κ light chain. Flow cytometry analysis showed that the 4H7 mAb bound to the L929/2IgB7-H3 and L929/4IgB7-H3 cells, whereas the 9C3 mAb did not bind to the L929/4IgB7-H3 cells, and only bound to the L929/2IgB7-H3 transfectants (Fig. 1). The competitive binding experiments also confirmed these conclusions. The data showed that one novel mouse anti-human 2IgB7-H3 specific mAb was established.

#### Expression of B7-H3 on different cell lines

The expression of 2IgB7-H3 and 4IgB7-H3 was examined in different human cell lines by flow cytometry. The data showed that B7-H3 expression in the majority of the cell lines could be detected by mAb 4H7. However, the expression of B7-H3 could only be detected in a few cell lines by the mAb 9C3 (Table I). This result suggested that the expression pattern of 4IgB7-H3 was more widespread than 2IgB7-H3 in the majority of the cell lines.

#### Expression of B7-H3 in human benign and malignant glioma tissues

**Figure 2.**
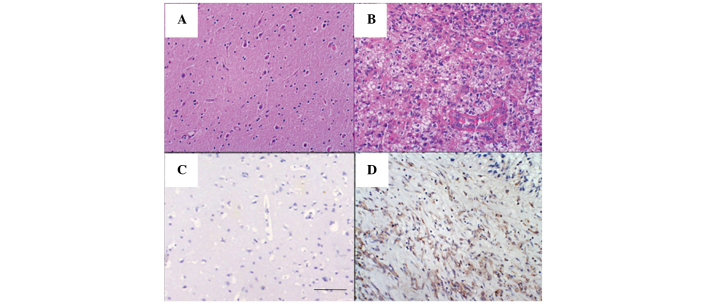
Immunohistochemistry analysis of B7-H3 in human normal brain and glioma tissues by 9C3. Expression of the 2IgB7-H3 isoform was found in glioma tissues, but not in normal brain tissues. (A) Normal brain tissue (H&E staining); (B) glioma (H&E staining; (C) normal brain tissue (9C3 immunostaining); and (D) glioma (9C3 immunostaining). Bar, 100 µm. H&E, hematoxylin and eosin.

**Figure 3.**
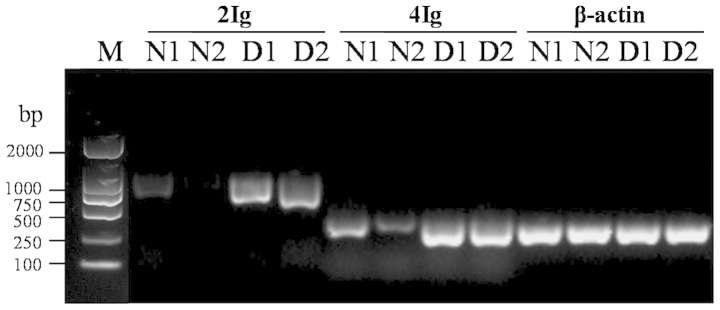
Reverse transcription-polymerase chain reaction detection of B7-H3 isoform expression in normal brain and glioma tissues. M, marker; N1 and N2, normal brain tissues, D1 and D2, glioma tissues; 2Ig, 2IgB7-H3; 4Ig, 4IgB7-H3.

Immunohistochemistry data revealed that 26 out of 35 glioma tissues were stained positive by 9C3, whereas the positive numbers detected by 4H7 were 27 out of 35 glioma tissues. No positive case was found in 10 normal brain tissues using 9C3. However, 6 positive cases were detected using 4H7 (Table II). These data indicated that 2IgB7-H3 is expressed in glioma tissues, but not in normal brain tissues (Fig. 2). By contrast, 4IgB7-H3 antigen is expressed at least in some human normal brain tissues. In addition, mRNA expression was analyzed by RT-PCR. The data revealed that 4IgB7-H3 mRNA was detected in the normal brain and glioma tissues. However, 2IgB7-H3 was found only in the glioma tissues. In normal brain tissues, 2IgB7-H3 mRNA expression was extremely weak or undetectable (Fig. 3).

## Discussion

B7-H3 (CD276) has been recently identified as a co-stimulatory member of the B7 family, and it shares 20–27% amino acid sequence homology with other members of the B7 family. B7-H3 is expressed in several non-lymphoid tissues of humans and mice, as well as in several human tumor cell lines. Expression can be induced by proinflammatory cytokines in monocytes and dendritic cells (1). The receptor of B7-H3 is located on activated T cells, but the nature of the receptor has not been identified. The functional role of the B7-H3 system remains controversial. Zhang *et al* detected high levels of the soluble form of B7-H3 (sB7-H3) in the sera of healthy human donors. The study found that sB7-H3 is released from monocytes, activated T cells, dendritic cells and certain carcinoma cells. Matrix metalloproteinase inhibitor can inhibit sB7-H3 release, with simultaneous increased membrane B7-H3 expression (18). The finding that sB7-H3 is bound to the B7-H3 receptor on activated T cells indicates that sB7-H3 has a functional role through the modulation of the B7-H3:B7-H3 receptor system.

B7-H3 has two isoforms in humans. The different expression pattern of the two isoforms (2IgB7-H3 and 4IgB7-H3) has not yet been reported. Therefore, the generation of mAbs that can discriminate 2IgB7-H3 from 4IgB7-H3 is of particular significance in understanding the expression and function of human B7-H3 isoforms. In the present study, a mAb, 9C3, was generated that specifically recognized 2IgB7-H3, but not 4IgB7-H3. By using mAb 9C3, the expression of 2IgB7-H3 was examined in a variety of cell lines. 2IgB7-H3 expression was detected in a few cell lines only, whereas 4IgB7-H3 was widely expressed in the majority of the cell lines. The regulation mechanism of this restricted expression of 2IgB7-H3 is not clear. The expression of B7-H3 isoforms in human glioma tissues was also examined and the expression of 2IgB7-H3 was found in the majority of the glioma tissues, but not in the normal tissues. Therefore, the detection of 2IgB7-H3 expression in glioma carcinoma tissues may be important to the assessment of the prognosis of glioma carcinoma patients and the choice of treatment.

In summary, one novel mAb recognizing 2IgB7-H3 was successfully generated in the present study. Using the mAb obtained, it was shown that 2IgB7-H3 is not the major isoform in a number of carcinoma cells. However, its overexpression in glioma suggests that 2IgB7-H3 mAb may be a useful tool for the diagnosis and therapy of glioma. Further studies are required to demonstrate the function of 2IgB7-H3 in glioma development.

## Figures and Tables

**Table I.** Expression of 4IgB7-H3 and 2IgB7-H3 in different cell lines, as determined by flow cytometry analysis.

Cell line	9C3 (2IgB7-H3)	4H7 (4Ig/2IgB7-H3)
Eahy926	++++	++++
HUVEC	–	++++
PODO	–	–
HK-2	–	++++
MC	–	+++
A549	–	++++
H1299	–	++++
SPCA-1	+	++++
H446	++	++++
H460	+++	++++
Colo-320	–	+++
CACO-2	–	++++
CW-2	–	++
LS174T	−/+	++++
SW480	−/+	++++
8266	–	++
U266	–	−/+
Daudi	–	–
Raji	–	–
HL-60	–	–
SHI-1	–	–
THP-1	−/+	++
Jurkat	–	–
U937	–	–
K562	–	–
HeLa	++++	++++
HO8910	+	++++
SK-BR-3	++++	++++
SIHA	+	++++
M435	+	++++
M231	+	++++
767	–	–
U251	++++	++++
SHG-44	–	–
Y79	++++	++++
AGS	–	++++
Hep-2	–	++++
HepG-2	+	+
SW1990	–	–
WI-38	+	+

−, ≥1 to <5%; −/+, ≥5 to <10%; +, ≥10 to <25%; ++, ≥25 to <45%; +++, ≥45 to <75%; and ++++, ≥75 to 100%.

**Table II.** Immunohistochemistry analysis of B7-H3 in human glioma tissues.

		9C3		4H7	
			
Tissue type	Total	Positive	Negative	Positive	Negative
Normal brain tissue, n	10	–	10	6	4
Glioma tissue, n	35	26	9	27	8

Positive, ≥10% brown cytoplasm; negative, <10% brown cytoplasm.
